# The effect of single-handed lifting tasks on the activation of the neck-shoulder shared musculature

**DOI:** 10.1080/23335432.2017.1296376

**Published:** 2017-03-06

**Authors:** Mohamed R. Amar, David Cochran, Jeffrey Woldstad

**Affiliations:** a Biomedical Engineering, University of Nebraska-Lincoln, Lincoln, NE, USA; b Industrial and Management Systems Engineering, University of Nebraska-Lincoln, Lincoln, NE, USA; c Biological System Engineering, University of Nebraska-Lincoln, Lincoln, NE, USA

**Keywords:** Electromyography, maximum voluntary contraction, shared musculature, cervical spine, musculoskeletal disorders

## Abstract

The use of the hand in lifting has been linked to occupational injuries of the neck and shoulder. This research is aimed at examining the effect of work-related factors on the major neck-shoulder shared musculature activity on both sides of the cervical spine for a right-handed lifting task. Subjects lifted different weights from 20 different locations produced by the interaction of varying heights, reach distance, and angles simulating the work done by assembly line workers. All lifting tasks were done by the right hand. Bilateral electromyography data of major shared musculature (upper trapezius, sternocleidomastoid, and levator scapula) were collected using surface EMG electrodes. Analyses demonstrate that work-related factors; hand weights, reach distance, angles, and gender significantly affect the activation level of active shared musculature of the neck. Results also showed that the active shared musculature (the right side) has a significant influence on the activation of the antagonistic shared musculature. The findings show that reducing the weights being handled and keeping work area closer to the body reduces the muscle activities in the shared muscles. These findings may be used to build a biomechanical model to predict the compressive forces acting on the cervical spine due to one hand lifting.

## 1. Introduction

The neck region is a common site of work-related musculoskeletal injuries and disorders (NIOSH 1997). Neck pain affects about 10% of the general population (Gore 1998). It is estimated that in as many as one-third of people, neck pain is not self-limiting and could progress to moderate long-term disability (Rothman 1982). In general, 30% of neck and cervical spine health problems among the working population are attributed to musculoskeletal injuries. The frequent sites of neck injury are C5 level (74%), C4 level (16%), and C6 level (10%) (Torg et al. 1991). The use of the upper extremities in working activities has been linked to neck musculoskeletal injuries. In work-related activities neck pain has been reported to be as high as 37% for food packers, 31% for cash register operators, 27% for office workers, and 63% for welders (Luopajarvi et al. 1979; Toner et al. 1991). It was concluded from epidemiologic studies that there is ‘evidence’ connecting forceful exertion of the arm and the occurrence of musculoskeletal injuries in the neck (Aaras & Ro 1997; NIOSH 1997). Lifting with one or both hands is one of the causes of the cervical-disc complex disorders (Kondo et al. 1981; Kelsey et al. 1984; Borenstein et al. 1998). A variety of occupations and/or work activities have been studied experimentally to understand the factors associated with the neck musculoskeletal disorders. EMG of the neck muscles was used to understand the mechanism of neck musculoskeletal disorders.

Harrison et al. (2009), used EMG to investigate neck muscle fatigue in the splenius capitis, sternocleidomastoid, and upper trapezius of helicopter pilots performing submaximal isometric endurance exertions. These experiments demonstrated significant levels of fatigue in the splenius capitis , and sternocleidomastoid, but no fatigue in the trapezius muscle. Nimbarte et al. (2010) evaluated the activities of the sternocleidomastoid and the upper trapezius muscles for subject workers performing isometric lifting tasks at different heights and in different neck postures (neutral, flexed, and extended neck postures). The results showed that EMG increased with both height and the magnitude of the exertion. The sternocleidomastoid muscle had the highest muscle activities during extended neck posture, while the upper trapezius had its highest during flexed neck posture. Nimbarte (2014) reports a similar study looking at gender differences in the response of the sternocleidomastoid and the upper trapezius muscles during different types of two-handed isometric lifts. This study found similar effects for exertion level and height. This study also reported significant gender effects with female subjects tending to rely more heavily on the sternocleidomastoid muscle to exert force as compared to male subjects.

EMG activities of infraspinatus, trapezius, and erector spinae muscles were studied on cash register operators in standing and sitting positions. Lannersten and Harms-Ringdahl (1990) studied the effect of four different cash registers on the EMGs of activities of infraspinatus, trapezius, and erector spinae muscles. Based on the pattern of EMG activities of the muscles studied, the authors concluded that keyboard and pen reader registers generated less EMG values than scanners in which the cashier needed to lift the product and scan it. In a similar study, Takala and Viikari-Juntura (1991) found that reducing the height of the service counter by 25 cm reduces the EMG amplitudes for the right upper trapezius of female bank cashiers. Dennerlein and Johnson (2006) measured muscle activity of four forearm muscles and three shoulder muscles for different positions of the computer mouse within computer workstations to evaluate biomechanical risk factors across different mouse positions. The three shoulder muscles monitored were the anterior deltoid, the medial deltoid, and the upper trapezius muscles. The forearm muscles studied were the flexor carpi ulnaris, flexor carpi radialis, the extensor carpi ulnaris, and the extensor carpi radialis. The high mouse position and tasks that have a mixture of mouse and keyboard usage resulted in the highest level of muscle activity of the shoulder muscles. Anton et al. (2005) studied construction workers in a laboratory setting to evaluate the effect lifting two different types of concrete blocks. Their results showed that the activity in the upper trapezius muscle activity was not affected by the block weight, but increased as the height of the wall increased. Lindberg et al. (1993) studied upper trapezius muscle activities for manual versus automated fabric-seaming tasks. The EMG amplitude analysis revealed a higher risk of musculoskeletal disorders for the manual seaming than the automated seaming tasks. Finsen et al. (1998) studied three of the most common dentistry work tasks. By evaluating EMG activity levels of splenius and upper trapezius muscles, all of the studied tasks showed high muscle activity levels. In a similar study, Pitts et al. (2005) used EMGs to evaluate 10 dentists. They found that EMG of the upper trapezius muscle revealed signs of fatigue at the end of an eight-hour shift. Nimbarte (2014) measured EMG of the sternocleidomastoid and the upper trapezius muscles during several different types of two-handed lifting tasks under 25, 50, and 75% exertion of their maximum strength. It was found that EMG magnitude of the upper trapezius as a percentage of maximum voluntary contraction (MVC) is higher than the percentage of MVC observed in the sternocleidomastoid. The authors concluded that both the load being lifted and the vertical position significantly affect the activation of these muscles. Females contract their sternocleidomastoid relatively higher than males.

While many of these studies found that the height and weight cause an increase in trapezius muscle activity during arm work, none have specifically studied the work layout factors on the activation of the shared musculature of the neck and shoulder that may cause injuries to the cervical spine and intervertebral discs. The objective of this research is twofold: (1) assess the effect of height, reach distance, weights, and angles on the activation levels of major muscles shared between the neck and shoulder regions above the C7/T1 level during one-handed lifting and (2) allow for an analysis of the co-activation counterpart muscles on the left side. While it has been found that hand lifting activities activate the shared muscles between the shoulder and cervical spine, suggesting an increase in the compressive forces on the cervical spine, biomechanical models proposed to estimate the compressive force on the cervical spine (Kumar & Scaife 1979; Moroney et al. 1988) do not include the activities which may lead to underestimation of compression. The results of this research might help in building a biomechanical model that can incorporate hand lifting activities to better estimate compressive forces. The muscles considered for this study were the left and right of sternocleidomastoid (SCM), upper trapezius (TRAP), and levator scapula (LEV). The sternocleidomastoid, upper trapezius, and levator scapula were chosen, in addition to being EMG accessible, because they are the major shared muscles between the shoulder and cervical spine. These muscles span the C7/T1 level, and have origin and insertion points both on the shoulder complex and the upper cervical vertebrae. The unique anatomical arrangement of these muscles suggest that their contraction may result in a compressive force acting on the cervical spine.

The lifts performed in this experiment were designed to simulate tasks known for resulting in neck problems. The main research hypothesis of this study was that work layout factors would significantly affect the activation levels of the shared musculature.

## 2. Method

### 2.1. Subjects

Ten subjects, five males, and five females, participated and gave their informed consent to the procedure, which was approved by the University of Nebraska-Lincoln Institutional Review Board. The mean height of the subjects was 170.8 (SD 6.01) cm, body weight 69.68 (SD 10.57) kg, and age 29 (SD 4.96) years. All subjects were right-handed and were screened for health history and were accepted only if they were without a history of back, neck, shoulder, arm, wrist, or hand pathology.

### 2.2. Experimental design

**Figure 1.**
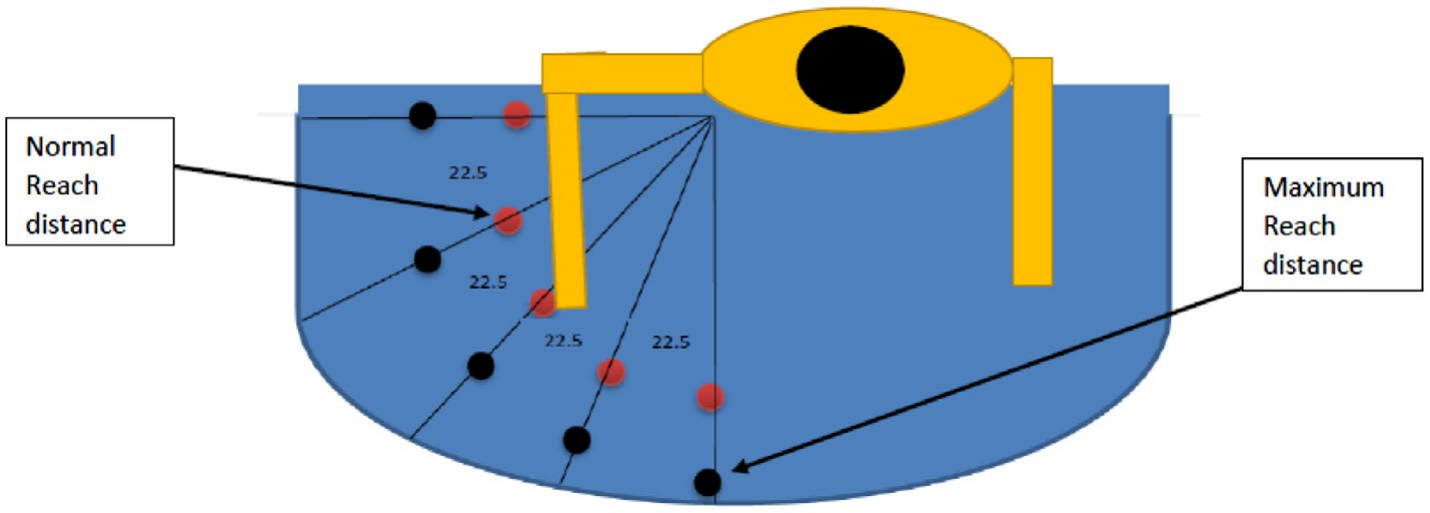
View of the distribution of loads.

**Figure 2.**
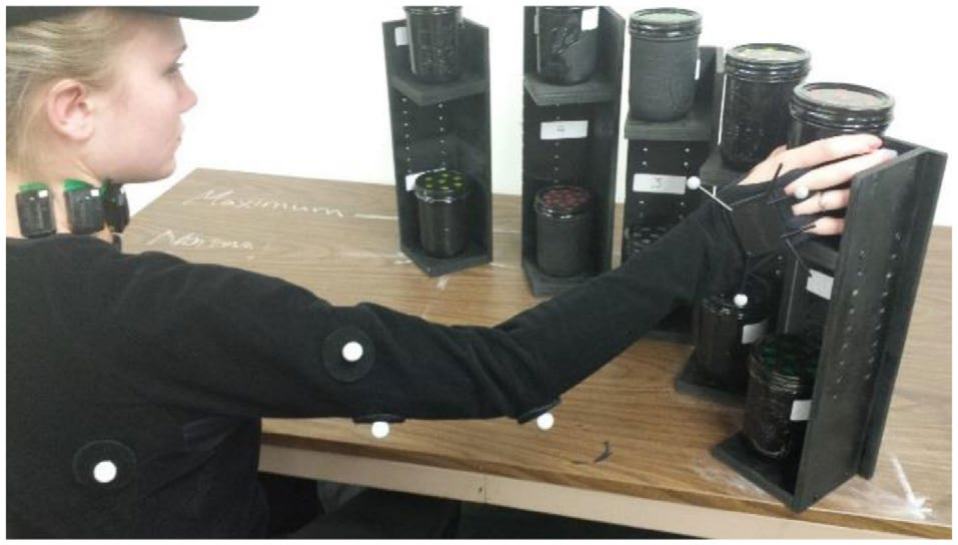
Participants performing lifting activities.

This study used a 5 × 2 × 2 × 5 design with the five weights, two heights, two reach distances, and five angles (distribution). Each subject lifted five different weights (1, 1.5, 2, 2.5, and 3) kg from 20 different locations. Those locations were the result of the interaction of two heights, two reach distances, and five angles. Therefore, there were five locations at the elbow height and the edge of the normal reach distance. Five locations were at the elbow height and the edge of the maximum reach distance. Five locations were at the shoulder height and the edge of the normal reach distance. Five locations were at the shoulder height and the edge of the maximum reach distance. Both the normal and maximum reach distances were determined based on the subject’s anthropometric data. Angles were measured from the edge of the work surface as: 0, 22.5, 45, 67.5, and 90 degrees. Each subject performed a total of 100 trials in random order. As a result, each muscle of the six muscles studied had a total of 100 responses. Subjects were allowed a resting period to eliminate the effect of fatigue. The experimental layout showing the heights, distances, and angles are shown in Figures 1 and 2.

### 2.3. Experimental task

Subjects who qualified to participate in the experiment were asked to complete personal data/medical history and consent forms at the beginning of their individual session. For each subject, anthropometric measurements and demographic data were obtained which included weight, height, upper arm length, forearm length, and hand length for the right arm. Only the right arm was used for the entire experiment. Initially subjects were given an opportunity to familiarize themselves with the lifting task of the experiment. After the familiarization session, they were asked (1) to wear an appropriate sized motion capture suit with reflective markers attached, (2) six surface EMG electrodes were placed on the upper trapezius, sternocleidomastoid, and levator scapula bilaterally. Three of these EMG electrodes were placed on the right side of the spine to measure the activation level of the active muscles and three were placed on the left side to measure the activation of the co-contraction of the counterpart muscles. The electrodes were placed using the technique of ‘best anatomical’ placement (Basmajian & DeLuca 1985). Verification of electrode placement was done using maximum voluntary isometric contractions (Harms-Ringdahl et al. 1986). Prior to attaching the electrodes, skin surfaces were first abraded and cleaned using isopropyl alcohol (Basmajian & DeLuca 1985).

After electrode placement had been verified, subjects performed the MVC required for normalization procedures (Granata & Marras 1993). MVC trials for each muscle were conducted before and after the experiment and the highest value was considered.

EMG values were normalized based on a MVC for each of the muscles in the study (Basmajian & DeLuca 1985; Harms-Ringdahl et al. 1986; Moroney et al. 1988; Granata & Marras 1993; Weaver 2006). A normalized EMG value was calculated as

$${\text{EMG}}_{\text{Normalized}}={\text{EMG}}_{\text{Task}}/{\text{EMG}}_{\text{Max}}$$

where EMG_Task_ was the measured filtered and integrated EMG value for a particular experimental trial, and EMG_Max_ was the highest normalization value based on a series of MVC trials for each muscle. The MVC normalization procedures were conducted consistent with a previous work by Moroney (1984) and Weaver (2006).

### 2.4. Testing procedures

Subjects were asked to sit in a chair and adjust the chair height until their elbows were parallel to the table surface. A foot rest was provided when needed. While seated, subjects were asked to lift the set of five weights of 1 kg (2.2 lb), 1.5 kg (3.3 lb), 2 kg (4.4 lb), 2.5 kg (5.5 lb), 3 kg (6.6 lb) from the 20 locations described above. These loads were randomly distributed on the table in front of the subject and they were lifted using just the right hand. The subjects were instructed to lift each weight slightly (about 2 inches) off the surface, maintain that for 2–3 s, and then place it back on the surface. While performing these lifting tasks, the EMGs and the posture data were recorded using the EMGs system and motion capture system respectively. A rest period of one to two minutes was provided between each trial with additional time upon request. After all trials had been successfully performed, the series of maximum voluntary muscle contractions were repeated.

### 2.5. Equipment

In this study, the EMG data was acquired using Trigno Wireless EMG system (Delsys Inc., Boston, USA). The system consists of a Trigno Personal Monitor that can transmit or store EMG data from 16 wireless electrodes. Eight OptiTrack Natural S250e cameras were positioned at the corners of the experimental area to form a cubic volume. These eight cameras were used to record posture data associated with the experiment. EMG signals were collected and initially amplified with a 10× gain setting in the electrode housing and sent to a main amplifier (total amplification was set at 1000×). Amplified signals were filtered using a band-pass filter, with cutoff frequencies of 20 and 450 Hz, and integrated. The band-pass filter was used to limit the overall bandwidth, improve the quality of the data, and allow for a more accurate time-history. The filtered signals were sampled at 1080 Hz. This sampling frequency was chosen because of the need to have motion capture and EMG data synchronized with respect to time. Motion capture data were sampled at a standard frequency of 60 Hz. Previous studies have shown a sampling rate of around 1000 Hz to be appropriate for this study (Basmajian & DeLuca 1985). The EMG sampling frequency of 1080 Hz was chosen as the largest multiple of 60 Hz close to 1000 Hz. All EMG data were collected using EMGWORKS Acquisitions and Analysis software (Delsys Inc., Boston, USA) and raw data were integrated on a time interval of 0.000926 s (1080 Hz) and a five-point (0.005 s) average around the peak was taken to smooth the data (Basmajian & DeLuca 1985).

### 2.6. Statistical analysis

The data were analyzed using a separate five-factor mixed-model analysis of variance (ANOVA) with subjects treated as a random effect (repeated measures) for each of the three active muscles. Within subject variables were height (2 levels) and hand loads (5 levels), reach distance (2 levels), angles (5 levels) while subject gender (2 levels) was between subjects. The dependent variables for ANOVA testing were the normalized EMGs values collected for three active muscles on the right side of the neck. Residuals of the data were plotted in a normal probability plot and were found to be approximately normal. Tukey Pairwise Comparisons were run on significant factors affecting the activation level of the active muscles to determine the change to which a muscle reacts significantly. Regression analysis was applied to determine how the active muscle influences its co-contracting counterpart. The dependent variable for the regression analysis was the normalized EMGs for the co-contracting muscle (on the left side of the spine) and the independent variable was the normalized EMGs of the active counterpart muscle. All analysis tasks were performed using the R computing environment.

## 3. Results

### 3.1. Analysis of variance

Results of the ANOVAs are presented in Table 1. It presents the effects of height, hand loads (weight), reach distance, and angles on the observed normalized EMG magnitudes (%MVC). All three active muscles showed a significant (*p* < 0.05) main effect for height, weight, reach distance, angles, and significant two factor interaction effect.

**Table 1.** Results of the separate ANOVA tests on the NEMG magnitude for the six muscles measured right sternocleidomastoid (SCM), upper trapezius (TRAP), and the levator scapulae (LEV).

Effect	Muscle								
	Right								
	TRAP			SCM			LEV		
	DF	*F*-Value	*P*-Value	DF	*F*-Value	*P*-Value	DF	*F*-Value	*P*-Value
Gender	1	65226.81	*	1	50537.89	*	1	9256.03	*
Weights	4	1519.52	*	4	865.75	*	4	1010.10	*
Heights	1	1427.21	*	1	872.20	*	1	2165.01	*
Reach distance	1	4209.88	*	1	2442.19	*	1	8160.60	*
Angles	4	517.64	*	4	300.18	*	4	1010.10	*
Gender × Reach distance	1	548.97	*	1	339.28	*	1	97.61	*
Gender × Height	1	167.70	*	1	118.25	*	1	31.78	*
Gender × Angle	4	67.83	*	4	41.43	*	4	11.78	*
Gender × Weight	4	188.80	*	4	121.92	*	4	32.99	*
Height × Reach distance	1	9.36	*	1	4.60	*	1	16.60	*
Height × Angle	4	2.40	*	4	8.5	*	4	3.82	*
Height × Weight	4	3.01	*	4	10.67	*	4	5.21	*
Angle × Reach distance	4	5.64	*	4	12.38	*	4	10.24	*
Angel × Weight	16	1.81	*	16	6.93	*	16	3.31	*
Weight × Reach distance	4	11.01	*	4	19.93	*	4	20.36	*

Note:

Stars indicated a *p*-value less than 0.05.

### 3.2. Tukey pairwise comparisons test

The results of Tukey Pairwise Comparisons presented in Table 2 show that a change of 1 kg will result in a significant increase in the level of activation in the upper trapezius and sternocleidomastoid muscles. However, a change of half a kg changes the level of activation in the levator scapula making the levator scapula more sensitive to hand load change. Table 2 shows that a change of 22.5 degrees or greater will result in a significant increase of the activation level in the upper trapezius, sternocleidomastoid, and levator scapula muscles respectively, if the position moves from the coronal plane to the sagittal plane. As was the case with the weights, the levator scapula shows more sensitivity to angles than the other two muscles. The sternocleidomastoid was less sensitive than the levator scapula but more sensitive than the upper trapezius.

**Table 2.** Tukey pairwise comparisons (means that do not share a letter are significantly different).

Muscles																				
UTAP							SCM						LEV							
Loads	*N*	Mean	Grouping				Loads	*N*	Mean	Grouping			Loads	*N*	Mean	Grouping				
3.0	200	33.113	A				3.0	200	23.868	A			3.0	200	37.683	A				
2.5	200	31.175	A	B			2.5	200	22.498	A			2.5	200	35.245		B			
2.0	200	29.303		B	C		2.0	200	21.117	A	B		2.0	200	32.840			C		
1.5	200	27.178			C	D	1.5	200	19.667		B	C	1.5	200	30.179				D	
1.0	200	52.236				D	1.0	200	18.302			C	1.0	200	27.735					E
Angles	*N*	Mean	Grouping				Angles	*N*	Mean	Grouping			Angles	*N*	Mean	Grouping				
90.0	200	31.522	A				90.0	200	22.741	A			90.0	200	35.679	A				
67.5	200	30.364	A	B			67.5	200	21.907	A	B		67.5	200	34.214	A	B			
45.0	200	29.166	A	B	C		45.0	200	21.085	A	B		45.0	200	32.697		B	C		
22.5	200	28.026		B	C		22.5	200	20.275	A	B		22.5	200	31.264			C	D	
1.0	200	26.927			C		1.0	200	19.445		B		1.0	200	29.827				D	

### 3.3. Regression

The relationship between the active muscle on the right side and the matching muscle on the left side referred here as the co-contracting muscle were tested using multiple regression. The dependent variables were the activation levels of the co-contracting muscles and the independent variables were the activation levels in the active counterpart muscles. Mathematical offshoots, including square root, square, cubic, and log of the independent variable, were included in the stepwise regression analyses. The stepwise regression results are shown in Table 3.

**Table 3.** Significant variables for co-contracting muscles.

	NEMG left UTAP	NEMG left SCM	NEMG left LEV
Variable	NEMG right UT	NEMG right SCM	NEMG right LS
*R*^2^	0.74	0.96	0.71

It can be seen in Table 3 that 74% of the variation of the activities of the left upper trapezius can be accounted for by the activities occurring in the square term of the right (active) upper trapezius. Likewise, it was found that 96% of the variation of the activities in the left SCM muscle is accounted for by the cubic term of the activities of the right SCM. Finally, Table 3 shows that 71% of the variation of the activities of the left levator scapula can be accounted for by the activation of the right levator scapula. The regression models for each of the significant variables found by the stepwise regression are presented in Table 4.

**Table 4.** Regression models for antagonistic muscles.

	Regression models for the co-contracting muscles	*R*^2^
NEMG left UT	5.245 + 0.689 (NEMG right Upper Trapezius)	0.74
NEMG left SCM	−1.325 + 0.61178 (NEMG of right SCM)	0.96
NEMG left LS	5.116 + 0.2694 (NEMG right Levator Scapula)	0.71

## 4. Discussion

The results demonstrated in Table 1 show that all factors studied were found to be significant to the contraction of the shared musculature. The results of the experiment are consistent with previous studies examining neck muscle activity in two-handed lifting tasks by Nimbarte et al. (2010) and Nimbarte (2014), even though the task investigated was quite different – initiation of a one-handed dynamic lift as opposed to a two-handed isometric exertion.

Justifications for the significant effect of height may be that at higher heights, subjects are required to generate more of the upward force vector through the shoulder muscles as opposed to torso extension. Also, it appears that lifting a heavier weight would increase the moment acting on the shoulder, which will require more muscle contraction to stabilize the shoulder joint. An explanation for the effect of reach distance may be that an increase in the distance results in an increase in the moment around the shoulder joint by increasing the moment arm, which leads to an increase in the muscle force to provide a counter moment required to lift the arm. In addition to the increase in the moment arm, changing the distance from normal to maximum requires extending the arm, which might affect the orientation of the muscles. This might result in the need for the shared musculature to exert more force to provide stability to the shoulder joint. Likewise, a change in the position of weights to be lifted (angle) can affect the moment around the shoulder and the anatomical orientation of the muscles, which might require more contraction of the shared musculature. Similar to Nimbarte et al. (2010), the results showed slightly different patterns of muscle activation for female subjects with somewhat higher activations than males. Generally, the cross-sectional area of muscles in females is relatively smaller than in males. The force generated by a muscle has a direct relation to its cross-sectional area (Cholewicki et al. (1995). Therefore, females may be required to contract their muscles relatively higher to produce the required forces.

Results demonstrated in Table 2 show that the muscles of the shared musculature react differently to the change of the factors that have more than two levels: the hand weights and the angles. The levator scapula showed a significant reaction to the change of both weights (half kg change) and angles (22.5 degrees change). The upper trapezius is relatively faster in sensing the change of angles (45 degrees change) than the sternocleidomastoid, but they both react with the same rate to the change of weights. Difference in muscle anatomical arrangement might justify this variation of sensitivity. The sternocleidomastoid is located in the anterior side of the neck while the hand loads create moment in the sagittal plan and the coronal plane pushing toward the transverse plane. This might suggest that the change of the moment is less sensible to the sternocleidomastoid. However, the levator scapula anatomical arrangement requires it to counter both moments toward the sagittal and coronal plane at the same time. This forces the levator scapula to react to the sum of the changes, and as a result, it would be more sensitive to the change of weights and angles. The upper trapezius is located on the posterior side of the neck making it reactive mostly to the change in the sagittal plane. Thus, it is less sensitive than the levator scapula.

Even though this was a right hand only task, it is interesting to find that the neck muscles for the left side of the neck were also contracting. The relation between the active shared muscles and their co-contracting counterparts is quite interesting. It appears that the activation of the active muscles has a significant effect on the contraction of their co-contracting counterparts (see Table 3). Regression analyses displayed in Table 4 showed linear relations between the active and co-contracting muscles. Co-activation of antagonist muscles has been demonstrated for the lumbar spine (Lavender et al. 1992; Marras & Mirka 1992; Granata & Marras 1995) and is thought to be related to the need for increased muscle stiffness or impedance (Bizzi et al. 1984; Selen et al. 2005). Given the importance of the head to human function, it is not unreasonable to suppose that impedance is also very important in the cervical spine. In addition, from a mechanical point of view, if the muscles of the right side of the neck are contracting to support a relatively large load applied to the right shoulder, a similar contraction on the left side of the neck is required to maintain an upright posture of the head.

Similar to the lower back, there is some evidence that physical work can lead to cervical disc prolapse (Grenady et al. 1993; Choi & Vanderby 1999; Côté et al. 2008). The muscles studied in this experiment (the sternocleidomastoid, upper trapezius, and levator scapulae), which span the cervical region of the neck, were found to be significantly affected by hand lifting activity. Therefore, these muscles contribute to the compressive force on the cervical intervertebral discs (Moroney et al. 1988). However, biomechanical models proposed to estimate the compressive force in the cervical spine (Kumar & Scaife 1979; Moroney et al. 1988) do not account for the increase in neck muscle force associated with lifting activities of the hands and may underestimate the compression in the cervical spine for these types of activities.

While results of this experiment show significant muscular activity associated with the single-handed lifting, it should be realized that the subjects participating in this experiment were university students, and differences may be found if experienced workers were used. This limitation has the potential to influence the external validity and occupational applicability of this research. Additionally, the results of this research show that the activation levels of the neck muscles are significantly affected by hand lifting. This supports the idea that an increase in the activation levels in these muscles increases the muscle force which may cause injuries to the cervical disc and other anatomical structures but not to the muscle itself. The dynamic task considered for this experiment was simplified to a single (static) point in time at the initiation of the lift. Analysis of the complete trajectory or consideration of different discrete points in the lift may have resulted in other conclusions. It is difficult to generalize these results to a dynamic task or for a repetitive lifting task. Additionally, our aim was to examine the effect of single-hand lifting tasks on the shared musculature between the neck and shoulder and to study the relation between the active and co-contraction muscles, and thus we designed our experimental task as such. Our analysis and results could further be used to build a comprehensive biomechanical model that is capable of taking into account the forces generated on the cervical spine as a result of hand usage.

## 5. Conclusion

In this research, the effect of arm usage on the shared musculature between the shoulder and the neck was studied. The single-hand lifting task studied in this experiment resulted in bilateral neck muscle exertions measured above the C7/T1 vertebral. EMG activities in the muscle studied were influenced by the layout factors and gender. This research revealed that the active contraction of the muscle studied could be used to predict the co-contraction of the counterpart muscles. This study also contributed to the mounting evidence showing that exertions involving the hands and arms have implications for work-related pain and injury in the neck region. The link between neck injury and hand exertions can often be overlooked due to the two-joint nature of this relationship. This research confirmed that a careful work layout design may prevent cervical spine injuries and cumulative disorders. Reducing the weights being handled and keeping the work area closer to the body can reduce the forces acting on the cervical spine. This research established the basis of building a comprehensive biomechanical model to predict the compressive force on the cervical spine.

## Disclosure statement

No potential conflict of interest was reported by the authors.
